# Supplementary material for: Impairment in quantitative microvascular function in non-ischemic cardiomyopathy as demonstrated using cardiovascular magnetic resonance
Source: PLoS One. 2022 Nov 18;17(11):e0264454. doi: 10.1371/journal.pone.0264454 (PMC9674167; doi:10.1371/journal.pone.0264454)
Supplement: S1 Table — (DOCX) [file pone.0264454.s001.docx]

| **Supplementary Table 1:** Comparison of rates of medical therapy utilization in NICM patients vs those with preserved LVEF. | | | |
| --- | --- | --- | --- |
|  | **Preserved LVEF**  **(N=58)** | **NICM**  **(N=41)** | **P-value** |
| ACE-Inhibitor/ARB n(%) | 30 (73%) | 15 (26%) | <0.0001 |
| Beta Blocker n(%) | 18 (31%) | 33 (80%) | <0.0001 |
| Mineralocorticoid Antagonists n(%) | 4 (7%) | 7 (17%) | 0.11 |
| Angiotensin Neprolysin Inhibitors | 0 (0%) | 0 (0%) | N/A |
| ARB, angiotensin receptor blocker; LVEF, left ventricular ejection fraction; NICM, non-ischemic cardiomyopathy. | | | |
