# Supplementary material for: Impairment in quantitative microvascular function in non-ischemic cardiomyopathy as demonstrated using cardiovascular magnetic resonance
Source: PLoS One. 2022 Nov 18;17(11):e0264454. doi: 10.1371/journal.pone.0264454 (PMC9674167; doi:10.1371/journal.pone.0264454)
Supplement: S2 Table — (DOCX) [file pone.0264454.s002.docx]

**Supplemental Table 1.** Multivariable gamma regression for predicting myocardial perfusion reserve

|  | ***B* coefficient**  **(95% CI)** | **SE (*B*)** | **P value** |
| --- | --- | --- | --- |
| NICM | -0.25  (-0.46 to -0.05) | 0.11 | 0.02 |
| Age | -0.007  (-0.02 to 0.0004) | 0.004 | 0.07 |
| White Race | -0.16  (-0.40 to 0.09) | 0.13 | 0.21 |
| Male Gender | 0.18  (-0.03 to 0.38) | 0.10 | 0.10 |
| Diabetes | -0.10  (-0.36 to 0.16) | 0.13 | 0.45 |
| Hypertension | -0.29  (-0.48 to -0.09) | 0.10 | 0.005 |
| LGE | 0.08  (-0.14 to 0.31) | 0.11 | 0.48 |
| NICM: non-ischemic cardiomyopathy; LGE: late gadolinium enhancement | | | |
