# Supplementary figures and images for: Impairment in quantitative microvascular function in non-ischemic cardiomyopathy as demonstrated using cardiovascular magnetic resonance
Source: PLoS One. 2022 Nov 18;17(11):e0264454. doi: 10.1371/journal.pone.0264454 (PMC9674167; doi:10.1371/journal.pone.0264454)

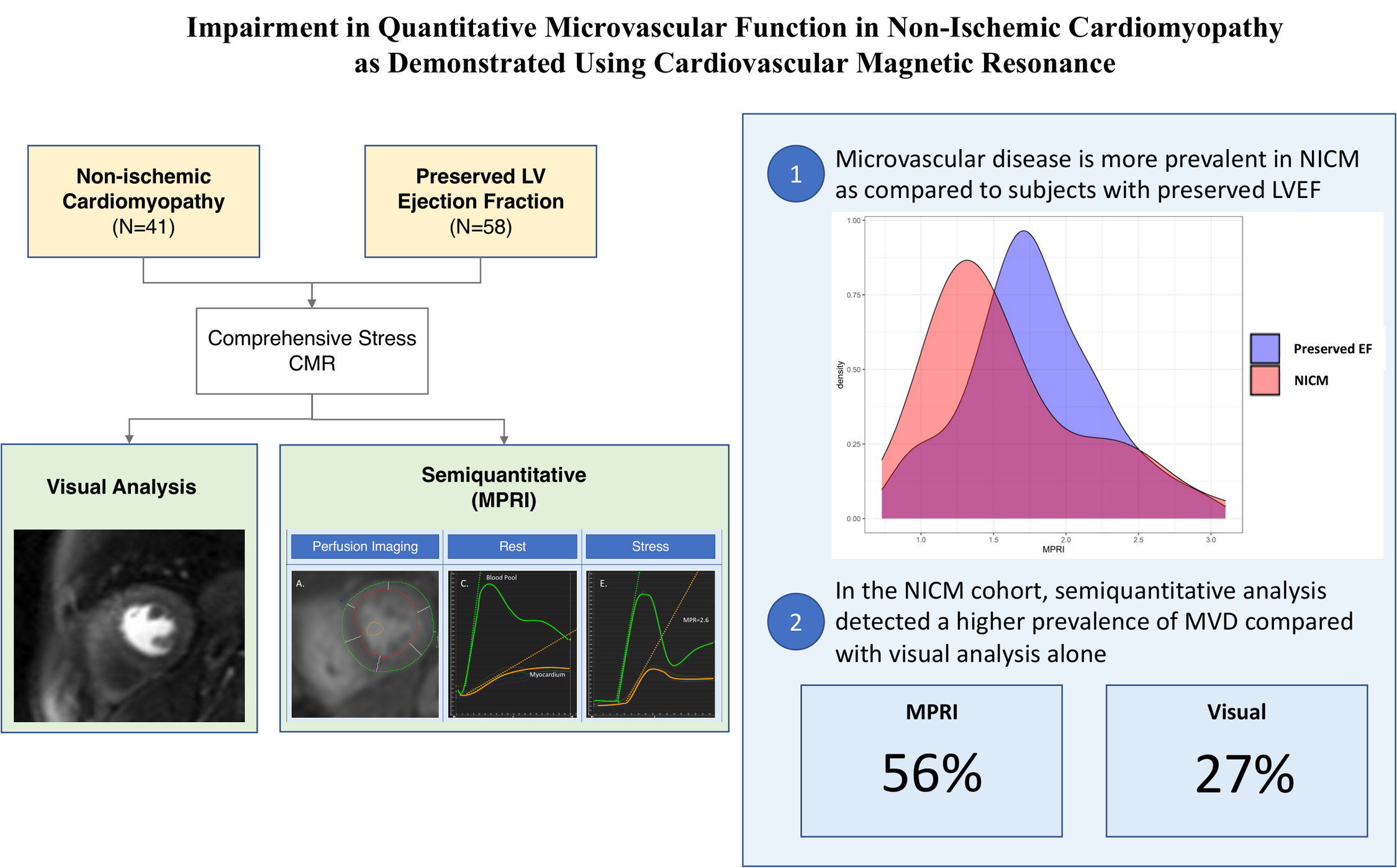

Supplement: S1 Graphical abstract — Left panel: study design and methods utilized to semi-quantitatively and visually assess microvascular disease (MVD) amongst patients with non-ischemic cardiomyopathy (NICM) and subjects with preserved left ventricular ejection fraction (LVEF). Right panel, Main findings: 1. Myocardial perfusion reserve index was significantly more impaired in NICM as compared to those with preserved LVEF. 2. Amongst those with NICM, MVD is detected significantly more often by semiquantitative as compared to visual methods. (TIF) [file pone.0264454.s003.tif]
