# Supplementary material for: Impairment in quantitative microvascular function in non-ischemic cardiomyopathy as demonstrated using cardiovascular magnetic resonance
Source: PLoS One. 2022 Nov 18;17(11):e0264454. doi: 10.1371/journal.pone.0264454 (PMC9674167; doi:10.1371/journal.pone.0264454)
Supplement: Attachment — Submitted filename: Response to Reviewers-MVD NICM-2022-6-14.docx [file pone.0264454.s004.docx]

**Response to Reviewers:** We thank the Reviewers for their thoughtful review of our manuscript entitled “*Impairment in Quantitative Microvascular Function in Non-Ischemic Cardiomyopathy as Demonstrated Using Cardiovascular Magnetic Resonance.”* We appreciate the opportunity to address their valuable feedback and look forward to the opportunity to further revise as necessary.

**Reviewer #1: The Authors assessed the prevalence of MVD in 41 NICM pts as compared with 58 controls, using stress CMR. MVD was defined visually as the presence of a subendocardial perfusion defect and semiquantitatively by myocardial perfusion reserve index (MPRI<1.51). NICM patients had lower rest slope (3.9 vs 4.9, p=0.05), lower stress perfusion slope (8.8 vs 11.7, p<0.001), and MPRI (1.41 vs 1.74, p=0.02). Visual analysis had a lower sensitivity in detecting MVD. The topic is interesting, but several points should be addressed.**

**Comment 1: One of the main limitation is the choice of control subjects, which appears as an heterogeneous group referred to CMR for several different reasons, and found (a posteriori) to have preserved LVEF (but 50-55% LVEF was included!), no LGE, no regional perfusion defects, no history of cardiac diseases. This is not a proper control group. In the control cohort, the indication for stress CMR was chest pain in 38 (66%), but it is unclear whether CAD was ruled out in these subjects. They still might suffer from microvascular angina, meaning that their MPRI is low because of an underlying cardiological disease.**

Response: We thank the Reviewer for this insightful feedback. On reflection, we agree with the Reviewer that ours is an imperfect control group as all patients were referred for clinical CMR examination and patients were not excluded on the basis of cardiovascular risk factors. Given this, we agree that it could be misleading to refer to the preserved LVEF group as a true control group. In response, we have revised the manuscript throughout to refer to these patients as “subjects with preserved LVEF” and not “controls.” Additionally, we revised the manuscript to better clarify exclusion criteria for the study. Lines 94-105 now state, “NICM was defined in accordance with guidelines by a clinical diagnosis of heart failure with LVEF ≤50% requiring loop diuretics and free of obstructive CAD (defined by the presence of >50% coronary artery stenosis on invasive or CT coronary angiography, prior myocardial infarction, prior percutaneous or surgical revascularization, or inducible epicardial ischemia)^16^. The preserved LVEF cohort was identified using consecutive patients referred for stress CMR examination between 2011 and 2016 free of heart failure, obstructive CAD, prior myocardial infarction, percutaneous or surgical revascularization. Those with a clinical diagnosis of heart failure—including heart failure with preserved ejection fraction, hypertrophic and/or infiltrative cardiomyopathies—and those requiring loop diuretics were excluded from the preserved LVEF group. Those with evidence of infarct scar (i.e. subendocardial or transmural) on CMR were also excluded from both cohorts. Using similar methodology to a recent study by Zhou *et al*, patients found to have a regional (i.e. non-circumferential) perfusion abnormality on stress CMR were excluded from both cohorts unless obstructive coronary artery disease was subsequently excluded by invasive or CT coronary angiography^17^.” We have additionally revised the limitations to reflect this (lines line 371-376), “Additionally, as patients in the preserved EF group were not excluded based on comorbidities, they cannot be viewed as a true “healthy control” group. However, this group is likely to be representative of the types of patients routinely seen in clinical practice. Additionally, because such patients were included, there were no significant intergroup differences in the prevalence of diabetes, atrial arrhythmias, and hyperlipidemia, thereby reducing the potential for confounding bias. We additionally attempted to address this by controlling for comorbidities in multivariable analysis.”

**Comment 2: In the control patients, focal perfusion defects on first pass stress perfusion was an exclusion criterion, which is quite unclear. How many patients had an isolated perfusion defects?**

Response: We thank the Reviewer for this insightful feedback. Patients with segmental (i.e. non-circumferential) perfusion defects were excluded from the study unless obstructive CAD was subsequently excluded by either invasive or CT coronary angiography. Based on these criteria, a segmental perfusion defect was only present in one patient in the NICM group in whom obstructive CAD was subsequently excluded by CT angiography. None of the patients in the preserved LVEF group had a segmental perfusion defect. We have revised the methods section (lines 102-105) to reflect this. Additionally, we have added the following (lines 226-228) to the results section to better clarify this point, “Only one patient in the NICM cohort had a segmental (i.e. non-circumferential) perfusion defect; in this patient, obstructive CAD was subsequently excluded by CT angiography. None of the patients within the preserved LVEF group had a segmental perfusion defect.”

**Comment 2: In the control patients, LGE was present in 16% of cases. What was the underlying disease? Might this impact on the microvascular function of these "normal" patients?**

Response: We agree with the reviewer that the presence of LGE might regionally affect microvascular function in both groups. For this reason, we specifically decided to control for the presence of LGE in multivariable analysis. To reflect this, lines 337-341 in the discussion now state, “Given the theoretical potential of LGE to affect regional microvascular function, we felt it important to control for the presence of LGE in multivariable analysis. MPRI remained significantly different between NICM and preserved LVEF patients even after controlling for the presence of LGE.”

**Comment 3: In the methods, it is unclear whether a single short axis or 3 short axis slices were used for qualitative and semiquantitative perfusion analysis.**

Response: Microvascular function was assessed using the mid short-axis slice. This was done for consistency as many of the distal short-axis slices could not be analyzed in the NICM cohort due to left ventricular wall thinning resulting in artifact related to partial volume effects. We have revised the methods to clarify this (lines 150-153), “First-pass perfusion imaging was performed in the 3 short axis slices using a 0.05 mmol/kg bolus of gadolinium. The mid-ventricular slice was chosen for analysis to maintain consistency and minimize artifact related to partial volume effects.”

**Comment 4: A second main limitation is the lack of clinical and prognostic data. Actually, the Authors described MVD in two cohorts of patients with two different methods, without any reference standard, any correlation with disease type and severity (biohumoral data? arrhythmic burden?) or prognosis. Please comment/extend your data.**

Response: We agree with the Reviewer that prognostic data would strengthen the manuscript. Unfortunately, given the size of our cohort and relatively low event rate, we did not have sufficient power to perform this analysis. This is acknowledged as a limitation of the manuscript (lines 378-380), “Additionally, given the size of our cohort, we were unable to assess whether the presence of MVD derived from stress CMR was associated with adverse outcomes as has been shown previously with cardiac positron emission tomography (PET)^2^.”

**Comment 5: In the conclusion, the Authors state that "Compared with visual analysis, semiquantitative analysis had a higher sensitivity for detecting impaired stress perfusion with CMR", but these thwo methods were not compared against a reference standard (PET? quantitative perfusion CMR?) to assess their real diagnostic accuracy. The Authors should revise this point, for example: "MVD by visual analysis was present in significantly fewer patients than MVD according to semiquantitative analysis...".**

Response: We thank the Reviewer for this feedback. We agree that the term sensitivity does not apply to our study given the absence of a gold standard. We have revised this throughout the manuscript particularly within the abstract and discussion sections.

**Comment 6-A possible confounding variable is represented by the relatively thinner ventricles in NICM patients, which might increase the incidence of dark rim artifacts and should be carefully excluded from analysis. Did the Authors check for this point? Was there a relationship between wall thickness and MVD? And between LV volume and MVD?**

Response: All images were reviewed by an advanced CMR reader to ensure that differences in MPRI amongst groups were not related to artifacts related to dark rim and/or partial volume effects. We clarified this in the methods by stating that (lines), “Additionally, stress perfusion images were also closely reviewed by an advanced level 3 CMR reader to ensure that areas of dark rim artifact were not included within the tracings.” Additionally, the Reviewer provides an excellent suggestion that we further ensure that intergroup differences were not related to differences in LV volumes or wall thickness. As suggested, we have performed this analysis and did not find any significant relationship between MPRI and indexed LV mass, LV end diastolic volume, septal, or posterior wall thickness. We have included this analysis in the methods, results, and discussion section to highlight this. Lines 339-341 now state, “Additionally, we did not identify a significant relationship between MPR and LV mass, volume, or wall thickness, suggesting that intergroup differences in MPRI are unlikely to be related to artifact related to dark rim or partial volume effects.”

**Reviewer #2: The Authors retrospectively studied patients with non ischemic cardiomyopathy (NICM) and 58 control patients with preserved systolic function who underwent stress cardiac magnetic resonance (CMR) between 2011-2016. Microvascular disfunction (MVD) was defined visually as presence of a subendocardial perfusion defect and semiquantitatively by myocardial perfusion reserve index (MPRI). MPRI was compared between groups using univariate analysis and multivariable linear regression. 41 patients with NICM (mean age 51 ± 14, 59% male) and 58 controls (mean age 51 ± 13, 31% 35 male) were identified. In the NICM group, MVD was present in 23 (56%) and 11 (27%) by semiquantitative and visual evaluation respectively. Compared with controls, NICM patients had lower rest slope (3.9 vs 4.9, p=0.05) and stress perfusion slope (8.8 vs 11.7, p <0.001) and MPRI (1.41 vs 1.74, p=0.02). MPRI remained associated with NICM after controlling for several parameters, such as gender, hypertension, ethnicity, diabetes, and late gadolinium enhancement. The Authors observed that MVD—as assessed using CMR—is highly prevalent in NICM when compared to control patients with preserved systolic function. They concluded that semiquantitative assessment is more sensitive for detecting MVD compared to visual methods alone. The study was well planned and the results are really interesting, The method is suggesting and promising, stimulating further studies for the assessment of MVD.**

**Comment 7: Minor concerns are related to statistical analysis: please specify pair or unpair Student’s t-test.**

Response: We are very gracious to the Reviewer for their kind feedback. With respect to the feedback regarding which type of t-test was used, it was the Student’s paired t-test. We have revised the manuscript accordingly (line 197).

**Reviewer #3: This study uses CMR to assess coronary microvascular deficiency in NICM patients. It is well done,simple and clear.**

Response: We thank the Reviewer for their kind feedback and thoughtful comments.

**There are some issues**
**Comment 8: Control group: it is not clear the control group population. It is very likely control group subjects were not healthy because, for example, in these patients LGE has been detected. The authors should select healthy subjects, almost without previous cardiovascular events, in the control group. Moreover, as the author stated, with regard the control group (line 91-92): Additionally, patients with infarct scar by LGE or focal perfusion defects on first pass stress perfusion were excluded from the control group. If we look at table, there are 9 subjects of the control group with LGE……….**

Response: Feedback regarding the “control group” was also raised by Reviewer 1. We agree and have revised the manuscript extensively. First and foremost, we have removed the term “control” from our manuscript and have instead referred to them as “subjects with preserved left ventricular ejection fraction” throughout the manuscript so as to avoid confusion. As described above in **comment 1**, we have thoroughly revised the methods section to better clarify exclusion criteria for the NICM and preserved LVEF groups (lines 94-105). Additionally, the Reviewer brings up an excellent point that 9 subjects within the control group had LGE despite the exclusion of patients with infarct scar. In all instances, LGE in our cohort was in a non-ischemic (i.e. mid-wall or epicardial) pattern. We have revised the methods sections to clarify that infarct scar was defined as being subendocardial or transmural (line 102). Additionally, lines 224-225 in the results section state, “In all instances, LGE patterns were in a non-ischemic distribution—either mid-wall or epicardial—as those with LGE in a subendocardial or transmural infarct pattern were excluded.”

**Comment 9: In the multivariate analysis, the authors did not include any kind of functional and morphological cardiac parameter. It is important to know the relationship between MPRI and EF, MPRI and LV volumes. Also, it could be interesting to know the relationship between regional wall motion analysis (WMSI) and MPRI. This is because WMSI is one of the main cardiac variables with prognostic relevance. The authors stated that low MORI is not related to other clinical variables, but it is very likely they are related to morphological and functional cardiac variables.**

Response: The Reviewer makes an excellent point which was also raised by Reviewer 1. In response, we have included additional analysis in which we assessed the relationship between MPRI and LV volumes, mass, thickness, and LVEF. In this analysis, MPRI was not significantly associated with indexed LV end diastolic volume, indexed LV mass, LVEF, septal or posterior wall thickness. This reassures us that intergroup differences in MPRI are unlikely to be solely related to LV structure or function. We have incorporated these findings into the manuscript as described in comment 6.

**Comment 10: In the discussion, the second main result, that is the higher diagnostic performance to detect MPR deficiency with semiquantitative method in respect to qualitative method is well known and cannot be a salient result of the study. The authors can mention this result but without highlighting it.**

Response: We thank the Reviewer for this feedback. As suggested, we have removed this point from the first paragraph of the discussion section.

**Comment 11: In the table 1, please report the value range of the indexed LVEDV. It is very likely that all NICM were not dilated.**

Response: We have revised table one as requested to include the value range for indexed LVEDV.

**Comment 12: Table 2: in this table the data are referred to all population and not to NICM patients with and without MPRI <> 1,51. In the text the authors wrote that in the table 2 are reported data of NICM patients only. Please, clear this point. However, it is more important to know the difference among NICM patient with and without MPRI < 1,51.**

Response: We thank the Reviewer for this suggestion. Accordingly, we have revised table 2 to only include patients with NICM (and not the preserved LVEF cohort). We have also similarly revised this in the results section (lines 259-261), “Those with impaired MPRI were more likely to be hypertensive as compared to those with normal MPI. There was a trend towards increased prevalence of female gender amongst those with impaired MPRI. There were otherwise no significant intergroup differences with respect to comorbidities or CMR parameters.”
