# Supplementary material for: Impairment in quantitative microvascular function in non-ischemic cardiomyopathy as demonstrated using cardiovascular magnetic resonance
Source: PLoS One. 2022 Nov 18;17(11):e0264454. doi: 10.1371/journal.pone.0264454 (PMC9674167; doi:10.1371/journal.pone.0264454)
Supplement: Attachment — Submitted filename: Response to Reviewers-MVD NICM-2022-10-4.docx [file pone.0264454.s005.docx]

**Response to Reviewers:** We thank the reviewer for his/her thoughtful review of our manuscript. We have strived to address the concerns raised by the reviewer regarding the manuscript; the comments have contributed to increased manuscript clarity and quality. Minor revisions were performed throughout the manuscript for typographical and grammatical errors. We appreciate the opportunity to address their valuable feedback and look forward to the opportunity to further revise as necessary.

Reviewer #1: The Authors performed a qualitative and a semiquantitative analysis of myocardial perfusion in 41 DCM patients compared with 58 patients with preserved EF. The manuscript is interesting and overall well written, even though there are some points to address:

We thank the Reviewer for his/her positive view of our work and the thoughtful comments and suggestions.

-In patients with preserved EF (16% of which presented LGE), the final diagnosis was not reported; please include in the text or in a table (how many normal scans? how many prior myocarditis? other diseases?)

We thank the reviewer for this advice. To better clarify this, we have added the following sentence (lines 166-168) “Within the NICM group, the etiology of cardiomyopathy was secondary to drug/toxin, genetic, hypertension, myocarditis, sarcoidosis, other, and idiopathic in 8 (20%), 3 (7%), 2 (5%), 2 (5%), 2(5%), 5 (12%), and 19 (41%) respectively (**Figure 2)**.” Additionally, this information is also displayed in **Figure 2** of the manuscript.

Was severe valvular heart disease an exclusion criterion? What about mitral valve prolapse? Please specify in the methods, or comment in the results if any of the patients had similar diseases (which might impact on myocardial perfusion).

We thank the reviewer for this valuable feedback. As the reviewer suggests, we did exclude those with severe valvular regurgitation or stenosis were excluded from both cohorts. To clarify this in the manuscript, the following statement has been added (lines 91-93), “Those with evidence of infarct scar (i.e. subendocardial or transmural) on CMR and those with severe valvular regurgitation or stenosis were also excluded from both cohorts.”

-There are no data about medical therapy, both in DCM and in patients with preserved EF; please include in a (supplemental?) table and comment in the text

The reviewer makes an excellent suggestion. Based on this feedback, we have gone back and collected rates of medical therapy between the two groups. Unsurprisingly, there was a higher rate of beta blocker and ACE/ARB utilization in the NICM group as compared to controls with LVEF. As suggested we have added a **supplementary** **Table 1** describing these findings in the results section. Additionally, we have added the following sentence to the discussion section (lines 266-268) “Unsurprisingly, rates of heart failure medication utilization—including ACE-inhibitors, angiotensin receptor blockers, and beta blockers—were higher in the NICM cohort; further studies are needed to better assess the impact of modern heart failure therapies on microvascular function in NICM.”

-There is no mention of some recent studies on quantitative myocardial perfusion in DCM patients (See for example Gulati et al, DOI 10.1016/j.jcmg.2018.10.032), which should be mentioned and briefly discussed in the manuscript.

We thank the reviewer for sending us this excellent resource. In response, we have added a new paragraph to the discussion comparing our studies. Lines 250-256 now state, “Our study parallels a growing body of evidence including most notably Gulati *et al* in demonstrating a strong link between NICM and MVD^2,25,26^. A notable strength of the Gulati *et al* study was the utilization of fully quantitative CMR perfusion^26^. Fully quantitative perfusion has the potential to offer highly accurate quantification of myocardial blood flow at both stress and rest with robust correlation with both invasive and PET-derived CFR^27,28^. In comparison to quantitative perfusion which requires a dedicated imaging sequence, semiquantitative techniques can be performed retrospectively on nearly any stress perfusion sequence, potentially allowing for more widespread availability.”
