# Supplementary material for: Impairment in quantitative microvascular function in non-ischemic cardiomyopathy as demonstrated using cardiovascular magnetic resonance
Source: PLoS One. 2022 Nov 18;17(11):e0264454. doi: 10.1371/journal.pone.0264454 (PMC9674167; doi:10.1371/journal.pone.0264454)
Supplement: Attachment — Submitted filename: Response to Reviewers-MVD NICM-2022-10-30.docx [file pone.0264454.s006.docx]

**Response to Reviewers:** We thank the reviewer for his/her thoughtful review of our manuscript. We have strived to address the concerns raised by the reviewer regarding the manuscript; the comments have contributed to increased manuscript clarity and quality. Minor revisions were performed throughout the manuscript for typographical and grammatical errors. We appreciate the opportunity to address their valuable feedback and look forward to the opportunity to further revise as necessary.

**Reviewer #1:**

**Comment 1:** The Authors addressed the comments and improved the manuscript; as highlighted in a previous comment, I would suggest the Authors to better specify the final diagnosis not only of the 41 NICM patients (which has now been provided), but also of the 58 control patients (16% of which presented LGE: how many normal scans? how many prior myocarditis? other diseases in the control patients?).

We thank the reviewer for this feedback. We have added the following sentence to the methods section (lines 96-97), “Additionally, those with disease-specific non-ischemic LGE patterns—such as myocarditis, sarcoidosis, or arrhythmogenic cardiomyopathy—were excluded from the preserved LVEF cohort.” Additionally, we have revised the results as follows (lines 186-188), “Among those in the preserved LVEF cohort in whom LGE was present, LGE was located in the insertion point in 7 (78%) patients; the remaining 2 (22%) patients had faint, non-specific inferolateral LGE.”
